# Proof-of-concept for effective antiviral activity of an *in silico* designed decoy synthetic mRNA against SARS-CoV-2 in the Vero E6 cell-based infection model

**DOI:** 10.3389/fmicb.2023.1113697

**Published:** 2023-04-20

**Authors:** Nofar Atari, Oran Erster, Yair Heskiau Shteinberg, Hadar Asraf, Eitan Giat, Michal Mandelboim, Itamar Goldstein

**Affiliations:** ^1^Central Virology Laboratory, Public Health Services, Ministry of Health, Sheba Medical Center, Tel HaShomer, Israel; ^2^Sackler School of Medicine, Tel Aviv University, Tel Aviv, Israel; ^3^The Department of Medicine, Sheba Medical Center, Ramat Gan, Israel

**Keywords:** SARS-CoV-2, antivirals, mRNA, *Betacoronavirus*, *in silico*

## Abstract

The positive-sense single-stranded (ss) RNA viruses of the *Betacoronavirus* (beta-CoV) genus can spillover from mammals to humans and are an ongoing threat to global health and commerce, as demonstrated by the current zoonotic pandemic of severe acute respiratory syndrome coronavirus 2 (SARS-CoV-2). Current anti-viral strategies focus on vaccination or targeting key viral proteins with antibodies and drugs. However, the ongoing evolution of new variants that evade vaccination or may become drug-resistant is a major challenge. Thus, antiviral compounds that circumvent these obstacles are needed. Here we describe an innovative antiviral modality based on *in silico* designed fully synthetic mRNA that is replication incompetent in uninfected cells (termed herein PSCT: parasitic anti-SARS-CoV-2 transcript). The PSCT sequence was engineered to include key untranslated cis-acting regulatory RNA elements of the SARS-CoV-2 genome, so as to effectively compete for replication and packaging with the standard viral genome. Using the Vero E6 cell-culture based SARS-CoV-2 infection model, we determined that the intracellular delivery of liposome-encapsulated PSCT at 1 hour post infection significantly reduced intercellular SARS-CoV-2 replication and release into the extracellular milieu as compared to mock treatment. In summary, our findings are a proof-of-concept for the therapeutic feasibility of *in silico* designed mRNA compounds formulated to hinder the replication and packaging of ssRNA viruses sharing a comparable genomic-structure with beta-CoVs.

## Introduction

Since the initial identification of severe acute respiratory syndrome coronavirus 2 (SARS-CoV-2) infections in China on December 2019 (Wu et al., 2020; Zhu et al., 2020), the ongoing pandemic of novel coronavirus disease 2019 (COVID-19) has placed a significant burden on health systems and economies (Richards et al., 2022). Up to date (February 2023) there have been > 750,000,000 confirmed cases of SARS-CoV-2 infections reported to the World Health Organization with more than 6,800,000 estimated deaths worldwide.^1^ The continuous emergence of multiple SARS-CoV-2 variants of concern raises the likelihood of further evolution of variants that escape existing vaccines and antivirals (El-Sadr et al., 2023).

Viral zoonoses are an ongoing threat to human health (Morens et al., 2004; Moya et al., 2004). The process of globalization and the expansion of international travel have made humanity vulnerable to zoonotic pandemics (Lloyd-Smith et al., 2009; Plowright et al., 2017). Noteworthy, the beta-CoV genus contains some highly pathogenic viruses that can spillover from mammals to humans (Graham et al., 2013; Fehr and Perlman, 2015; Perlman, 2020). Beta-CoVs are enveloped positive-sense ssRNA viruses with relatively large genomes ranging from 28 to 32 kb (Fehr and Perlman, 2015). In the past 20 years three outbreaks of zoonotic pathogenic beta-CoVs have evolved (Perlman, 2020), including the first human SARS-CoV (Falsey and Walsh, 2003), Middle East Respiratory Syndrome (MERS) coronavirus (Zaki et al., 2012), and recently SARS-CoV-2. The latter virus is responsible for COVID-19, which is respiratory illness ranging from “common cold” symptoms to a severe acute respiratory disease combined with an excessive immune response culminating in a life-threatening cytokine storm (Laing et al., 2020).

Contemporary anti-viral therapies focus on vaccination and targeting key viral proteins with specific monoclonal antibodies or small-molecule drugs (Santos et al., 2020; Shyr et al., 2020). However, as turned out to be evident during the ongoing COVID-19 pandemic, it took almost a year into the pandemic to develop and distribute effective vaccines (Polack et al., 2020; Baden et al., 2021), meanwhile the spread of COVID-19 was exponential and produced a devastating toll on global health and the economy.

The mRNA-based vaccines developed by Pfizer-BioNTech (COMIRNATY) and Moderna (SPIKEVAX) have initially proven to offer effective protection from severe disease caused by the wild type (WT) variant of SARS-CoV-2 (Polack et al., 2020; Baden et al., 2021; Bar-On et al., 2021). However, it has been realized that this protection wanes over time and the level of neutralizing antibodies of post-vaccination sera declined significantly over the first six-month (Levin et al., 2021). Likewise, the neutralization efficiency of post-vaccination sera against emerging SARS-CoV-2 variants of concern including the B.1.351 (beta), B.1.617.2 (delta), and B.1.1.529 (omicron) was significantly lower compared to the SARS-CoV-2/Wuhan-Hu-1 WT-strain (Nemet et al., 2022). Accordingly, clinical data confirmed that two doses of vaccine provided only a limited protection against symptomatic disease caused by the omicron variant of concern and that third and fourth booster doses were needed to significantly increase protection, but even this protection decreased significantly after 3 months (Regev-Yochay et al., 2022; Tartof et al., 2022).

Thus, the COVID-19 pandemic, exposed the unmet need for an unconventional, relatively simple to produce and adaptable, antiviral modality that can be made available at an early phase of a new zoonotic viral outbreaks. Importantly, the complete genome sequence of the novel SARS-CoV-2 was discovered relatively soon after the outbreak of COVID-19 (Wu et al., 2020). Consequently, information from previous and new research, encompassing virus reverse genetics and secondary structure modeling studies of viral RNA genomes, could be used to delineate evolutionary conserved cis-acting regulatory RNA elements (CREs) within the SARS-CoV-2 genome that are vital for its replication and packaging (Hsieh et al., 2005; Stobart and Moore, 2014; Yang and Leibowitz, 2015; Lim and Brown, 2018; Masters, 2019; Alhatlani, 2020; Rangan et al., 2020).

In this context, the spontaneous generation of “truncated” defective viral genomes (DVGs) during the replication of RNA viruses, by the error-prone viral replicase-transcriptase complex, is a rather common occurrence. However, the capacity of naturally created DVGs to interfere with standard viral genome replication and encapsidation and how they influence the viral disease outcome are unclear. The *in vitro* relevance of DVGs may depend on their sequence length, production level, and the ratio of DVGs to standard viral genomes within confined intracellular hubs of infection (Tapia et al., 2013; Genoyer and López, 2019).

Our working hypothesis was that these insights can now be applied to the *in silico* design of a synthetic-mRNA-based rational therapy. The decoy mRNA sequence should contain all critical genomic CREs and obviously be replication incompetent within uninfected cells. In particular, it must have a meaningfully shorter sequence compared to the standard viral genome to efficiently compete for replication. Consequently, at the outset of the Covid-19 pandemic, we engineered a short ∼1500nt-long synthetic mRNA, termed PSCT, and submitted a US provisional patent application (US 63/008,756) on April 12, 2020, providing the details of the PSCT invention. In brief, The PSCT contained the following genomic sequences from SARS-CoV-2/Wuhan-Hu-1WT strain (NC_045512.2): an extended 5′UTR required for genome replication by the viral replicase-transcriptase complex; a predicted genome packaging signal (PS); and a highly conserved 3′UTR sequence.

Here we show, using the *in vitro* model of Vero E6 cells infected with a SARS-CoV-2 WT-isolate that treatment of the cell cultures with PSCT, encapsulated in liposomes, significantly inhibited SARS-CoV-2 genome replication as well as viral particles release from infected cells into the extracellular milieu.

## Materials and methods

### Virus isolation titration and storage

All assays involving potentially infectious SARS-CoV-2 were performed in a Biosafety Level 3 laboratory (BSL-3). The WT SARS-CoV-2 virus was formerly isolated from nasopharyngeal samples, as detailed in previous publications (Lustig et al., 2021; Nemet et al., 2022). Briefly, by next generation sequencing we identified nasopharyngeal samples from SARS-CoV-2 positive individuals that contained the Wuhan sub lineage B.1.1.50 (hCoV19/Israel/CVL-45526-ngs/2020). Confluent Vero E6 cells were incubated for 1 hour at 33°C with 300 μL of nasopharyngeal samples followed by addition of Minimum Essential Medium (MEM) Eagle supplemented with 2% fetal calf serum (FCS). Upon cytopathic effect (CPE) detection, the supernatants were aliquoted, and virus titration and storage at −80°C were done, as previously described (Nemet et al., 2022). This protocol was previously approved by the Institutional review board of the Sheba Medical Center (approval number: SMC-8008-20).

### Analyzing antiviral activity of PSCT on SARS-CoV-2 replication in Vero E6 cells

#### Cell cultures

Trypsinized Vero E6 cells were suspended in MEM-Eagle 10% FCS and plated at 2 × 10^5^ cells per well in 24-well plates. The following day the cells were visualized using a light microscope, and we proceeded with SARS-CoV-2 infection once the cells had reached appropriate confluence.

#### Preparation of liposome encapsulated mRNA

Prior to transfection we prepared mRNA/liposome complexes using lipofectamine-MessengerMAX™ transfection reagent as recommended by the manufacturer (Thermo Fisher Scientific Inc.).

#### Infection

Culture media was gently removed and cells were infected with the WT SARS-CoV-2 at a low multiplicity of infection (MOI) of 0.01 for 1 h at 33^°^C. Next, the cells were washed and the medium was replaced with 0.5 ml of MEM-Eagle 2% FCS, as previously detailed (Nemet et al., 2022).

#### PSCT transfection

At 1 h post infection (p.i.), the designated cultures were treated with the PSCT/lipid complexes prepared beforehand, as follows: final lipofectamine-MessengerMAX™ volume of 1.5 μL premixed with 125, 250, or 500 ng of PSCT per well (done in 24-well plates). The assay plates contained equivalent triplicate wells with infected cells either treated with lipofectamine only (mock transfection) or left untreated (negative control). Four hours after transfection, the culture medium was aspirated and the adherent cells were washed twice with cold MEM. We then added 0.5 ml of fresh MEM-Eagle 2% FCS and maintained the infected cell cultures in a humidified incubator (33°C and 5% CO_2_). The supernatants and/or cells were collected at various time points, as indicated, and used for downstream analysis. Each experiment was done in triplicate and repeated at least three times.

### Monitoring cell culture viability by crystal violet dye staining

Cell cultures plates were observed daily with an inverted optical microscope by two skilled operators for confluence and viability. As adherent cells detach from cell culture plates during cell death, this occurrence can be used for the indirect quantification of cell death due to toxicity or viral cytopathic effects by staining the adherent cell cultures with the crystal violet dye that binds proteins and DNA (Feoktistova et al., 2016). Thus, we used this method to assess the effect of PSCT on cell viability. Briefly, at the end of cell culture experiments, the supernatants were removed and cells were fixed with 4% formaldehyde, stained with 0.1% crystal violet solution for 2 h at room temperature, evaluated with an inverted optical microscope, and recorded by digital imaging.

### Viral RNA genome and PSCT quantification

Total RNA was extracted from supernatants or cell lysate of indicated Vero E6 cell using the MagDEA DX SSV kit (Precision System Science Co., Ltd., Japan) on the MagLead 12gC platform, according to the manufacturer’s instructions (Erster et al., 2021). Equal amounts of purified total RNA were then analyzed by one-step reverse transcription quantitative real-time PCR (qPCR) using the SensiFast one-step probe mix from Bioline GmbH,^2^ according to the manufacturer’s instructions. In order to distinguish between the viral RNA genome and the PSCT, we used a duplex qPCR assay containing two reactions, one targeting the viral E gene and one targeting the synthetic transfected mRNA. The SARS-CoV-2-specific reaction specifically targeting the viral E gene was previously described by Corman et al. (2020). The PSCT-specific reaction was designed to target the junction between the stop codon repeats and the PS regions that forms a sequence absent from the viral genome. To avoid non-specific amplification by the PSCT-specific qPCR reaction we performed bioinformatics analysis to exclude off-target sequence detection within the SARS-CoV-2 and Chlorocebus Sabaeus genomes. The amount of the PSCT molecules in each sample was determined using a linear regression formula of the standard curve obtained from testing serial dilutions of the PSCT in RNase-free deionized water.

The PSCT-specific primers and probes were as follows:

PSCT forward primer: 5′-AAAAGCCGTTTTGCCTCAAC-3′
PSCT reverse primer: 5′-TGTCCATCAAAGTGTCCTATC-3′
PSCT probe: 5′-FAM/CAGCCCTATGTGTTCTAGTAAATG/BHQ1-3′

### Statistical analysis

Statistical analysis was performed in the R language and environment for statistical computing and graphics, software version 4.0.4, URL: https://www.r-project.org/. The statistical significance testing of PSCT treatment effects was evaluated by various tests, as follows: the linear mixed effects (LME) modeling with the random effect correction procedure, the simple linear regression modeling to compute the adjusted coefficient of determination (r-squared), and the Student’s t-test. P values < 0.05 were considered statistically significant.

## Results

### *In silico* design of the PSCT sequence and manufacturing of the synthetic mRNA

The beta-CoV genomes contain conserved cis-acting RNA sequences forming unique secondary structures vital for viral replication (Stobart and Moore, 2014; Fehr and Perlman, 2015; Yang and Leibowitz, 2015; Masters, 2019; Sanders et al., 2020). Hence, to construct the PSCT sequence, our key aim was to identify the evolutionary conserved CREs that are obligatory for SARS-CoV-2 genome replication and packaging, and that are unlikely to harm uninfected host cells.

Beta-CoV genomes have been postulated to contain a cis-acting PS sequence that triggers genome packaging and regulates viral capsid assembly (Masters, 2019). Thus our next goal was to identify a comparable PS sequence within the novel SARS-CoV-2 genome. Pertinently, Chang and colleagues (Hsieh et al., 2005) have studied the mechanisms involved in the assembly and genome packaging of the former first SARS-CoV (NC_004718.3). Initially, they performed RNA secondary structure analysis and identified a distinct and conserved 63-nt stem-loop structure (termed PS63) in the 3′ end of the ORF 1b of SARS-CoV (nucleotides 19888 to 19950). Next, by establishing an *in vitro* system to produce virus-like particles (VLPs) in Vero E6 cells, they demonstrated that a cDNA fragment encompassing the SARS-CoV genome from nucleotides 19715 to 20294 (termed PS580) and includes PS63 could be efficiently packaged within the VLPs.

**FIGURE 1:**
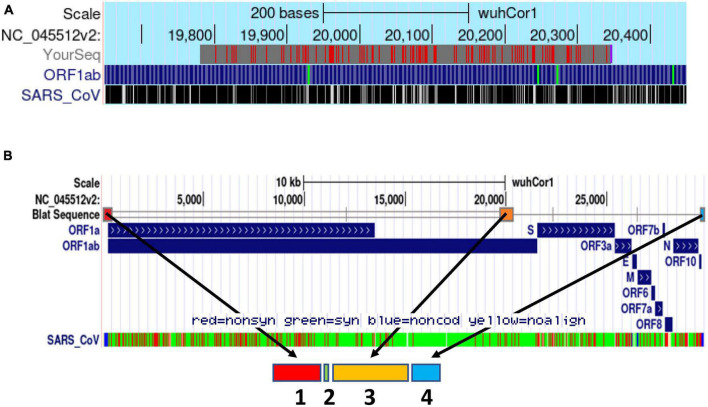
Schematic delineation of the *in silico* design of the synthetic PSCT mRNA. **(A)** Alignment of PS580 sequence of SARS-CoV (position 19715 to 20294) and the SARS-CoV-2 genome (NC_045512.2) using the SARS-CoV-2 BLAT online tool developed by the UCSC Genome Browser Group (red marks: genome and query sequence have different bases at this position). **(B)** Illustration of the genomic loci of the PSCT mRNA sequence modules within the genome of SARS-CoV-2 using the BLAT search online tool. As indicated by the arrows, the synthetic PSCT was comprised of three relevant conserved CREs (sequence module 1, 3, and 4) and a short artificial sequence of various stop codons (module 2) inserted amidst the first two CRE modules. In addition, as shown in the lowermost portion of panel **(B)**, we performed in parallel sequence alignment of the PSCT modules and the SARS-CoV isolate TW1 genome (green: synonymous, red: non-synonymous nucleotide substitution, and blue non-coding sequences).

Based on this report, we hypothesized that the PS of SARS-CoV-2 might be located in a conserved corresponding region at the 3′ end of its ORF1b locus. Thus, we performed pairwise sequence alignment of the PS580 sequence of SARS-CoV (NC_004718.3) and the WT SARS-CoV-2 genome (NC_045512.2). First, using the SARS-CoV-2 BLAT online tool developed by the UCSC Genome Browser Group,^3^ we identified a sequence of >80% similarity (81.9%) in the in the 3′ end of the ORF 1b of SARS-CoV-2 spanning from nucleotides 19,781–20,346 (Figure 1A).

Next, we performed a multiple sequence alignment of the predicted PS sequence of SARS-CoV-2 with nine relevant bat SARS-like-CoV genomes and the first human SARS-CoV using the Geneious software (Version 9.18, Biomatters, Inc.). We found an overall high sequence identity (> 85%) at this genomic region (Supplementary Figure 1A). These observations, supported our notion that this PS580-equivalent genomic region may indeed represent a functional PS within the SARS-CoV-2 genome.

Many studies conducted prior to the current COVID-19 outbreak, as reviewed by Yang and Leibowitz (2015), have suggested that highly conserved and functional CREs were located in the 5′UTR and the adjacent ∼200 residues of the first open reading frame (ORF1a) as well as within the 3′UTR sequence. Thus, to assess the level of conservation of the “extended” 5′UTR among SARS-like coronaviruses we aligned the first 475 nucleotides of the wild-type SARS-CoV-2, the first human SARS-CoV and of nine relevant bat SARS-like-CoVs (Geneious software). This, analysis (Supplementary Figure 1B) verified an overall strong sequence identity (>90%) at this 5′ genomic sequence, supporting our working hypothesis that this extended 5′UTR serves as a key CRE in beta-CoV genomes vital for its replication and translation.

Furthermore, by multiple sequence alignment (as described above) we assessed the sequence conservation of a 3′UTR sequence (positions 29,606–29,876) predicted to contain conserved CREs forming secondary and higher-order structures vital for genome replication (Yang and Leibowitz, 2015; Manfredonia et al., 2020; Rangan et al., 2020, 2021). This analysis revealed a very significant sequence identity (> 95%) among SARS-like-CoV genomes in this 3′ end locus (Supplementary Figure 1C).

Based on these analyses, we designed and patented the “decoy” PSCT mRNA that included the three relevant genomic CRE sequences of SARS-CoV-2 isolate Wuhan-Hu-1 (NC_045512.2). As shown in Figure 1B, the 5′-end of our transcript covered the extended 5′UTR (positions 1–475), followed by the *in silico* predicted genome PS (positions 19,657–20,384), and a highly conserved 3′-end sequence (positions 29,603–29,870). In addition, to prevent aberrant protein translation, the extended 5′UTR sequence was flanked at its 3′-end with a short sequence (17 nucleotides) containing various translation termination stop codons (see Supplementary data 1).

Highly efficient *in vitro* transcribed (IVT) mRNA technologies have evolved in recent years that permit rapid manufacturing of potentially therapeutic synthetic mRNAs (Karikó et al., 2005; Sahin et al., 2014; Pardi et al., 2015). Accordingly, the PSCT product was specially synthesized by TriLink BioTechnologies (San Diego, CA, USA), URL: https://www.trilinkbiotech.com/custom-mrna-synthesis. First, a refined proprietary method of solid-phase chemical synthesis of the relevant DNA oligonucleotide sequence was employed to obtain the core sequence of the PSCT. Next, the linearized DNA sequence was used as a template to produce RNA via T7 RNA-polymerase-dependent synthesis. The proprietary co-transcriptional capping was achieved by the CleanCap^®^ technology yielding high quality capped mRNA. This customized *in vitro* RNA transcription yielded a 1569nt-long capped and polyadenylated mRNA. The PSCT product was provided after passing a rigorous quality control process by the manufacturer.

### Transfection with liposome-encapsulated-PSCT significantly inhibits SARS-CoV-2 replication in Vero E6 cells in a dose-dependent manner

**FIGURE 2:**
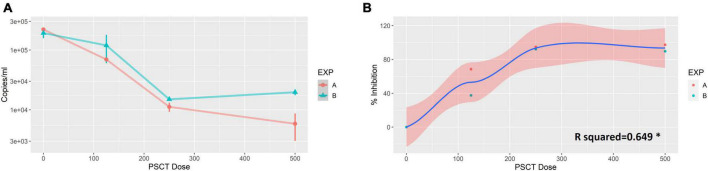
Dose-dependent inhibition of SARS-CoV-2 replication in Vero E6 cells by liposome-encapsulated-PSCT treatment. Vero E6 cells were plated in 24-well plates and infected with SARS-CoV-2 viral stock at a low MOI. At 1 h p.i., the virus inoculum was aspirated, and corresponding cultures were treated with three serial dilutions of liposome-encapsulated-PSCT (500, 250 and 125 ng of PSCT per well), as detailed in the results section. **(A)** The supernatants were collected at 24 h p.i., and SARS-CoV-2 RNA load in the cell culture supernatant was determined by qPCR (genome copies/ml). **(B)** Depicting the positive correlation between PSCT dose and % inhibition of viral genome load in supernatants collected from the indicated treatment groups, as compared to mock treatment group (R squared = 0.649 by the linear regression model, *p < 0.01). Data shown represent the mean ± SEM of two independent experiments, marked as EXP **(A,B)**, done in triplicates.

Vero E6 cells were plated at 0.1 × 10^6^ cells per well in 24-well plates and visualized 24 h later using a light microscope to confirm that the cultures reached the appropriate confluence of ∼90%. Next, the cells were infected with SARS-CoV-2 viral stock at a low MOI of 0.01. At 1 h p.i., the virus inoculum was aspirated, and corresponding cultures were treated with three serial dilutions of liposome-encapsulated-PSCT (500, 250, and 125 ng/well). The supernatants were collected at 24 h p.i., and SARS-CoV-2 viral load in the cell culture supernatant was determined by qPCR (genome copies/ml). Our results, as depicted in Figures 2A, B, revealed that transfection with liposome-encapsulated PSCT induced a significant dose-dependent inhibition of SARS-CoV-2 replication in the designated treatment groups as compared to the mock transfection group (R squared = 0.649 by linear regression model, *p* < 0.01). For example, transfection of infected Vero E6 with the highest dose of liposome-encapsulated PSCT (500 ng per well) significantly inhibited SARS-CoV-2 replication and consequent extracellular release compared to the mock treated cultures (∼94% on average, *p* < 0.0001 by the Student’s *t*-test). Whereas, transfection with 125 ng of liposome-encapsulated PSCT produced a smaller inhibitory effect as compared to mock treated cultures (∼53% inhibition, *p* < 0.01).

### PSCT transfection inhibit SARS-CoV-2 replication kinetics up to 72 h p.i.

Next, we studied the effect of liposome-encapsulated PSCT treatment on the replication kinetics of SARS-CoV-2 over time. Thus, Vero E6 cells were infected with SARS-CoV-2 viral stock at low MOI (0.01) for 1 h, as detailed above, and the cultures were then treated once with liposome-encapsulated PSCT (250 ng per well), mock treatment, or left untreated. At 4 h post transfection, we aspirated the media and replaced it with standard culture media (repeated two times). The supernatants and cell lysates were collected at 24, 48, and 72 h p.i., and used for downstream analysis to determine SARS-CoV-2 replication kinetics and reciprocal PSCT levels by multiplex qPCR.

**FIGURE 3:**
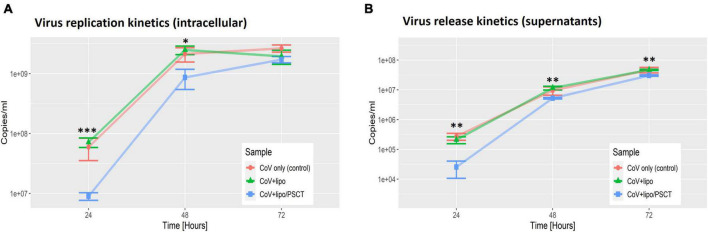
SARS-CoV-2 replication is decreased at 24–72 h p.i. by PSCT transfection as compared to mock treatment. Vero E6 cells were infected with SARS-CoV-2 viral stock at low MOI and the cultures were treated with liposome-encapsulated PSCT (250 ng/well), mock treatment, or left untreated, as detailed in the results section. The cell lysates **(A)** and supernatants **(B)** were collected at 24, 48, and 72 h p.i., and used to determine SARS-CoV-2 genome copies/ml by multiplex qPCR. **(A)** Depicting SARS-CoV-2 intracellular replication inhibition in the PSCT treatment group as compared to mock treated and untreated control cultures at the various time points. **(B)** Depicting significant inhibition of viral genome release into the cell-culture supernatants by PSCT treatment during the first 72 h p.i., as compared to mock treatment (*p* < 0.01). Data shown represent the mean ± SEM of one representative experiment out of three performed in triplicates. Statistical analysis was performed by the LME model (*p < 0.05, ^**^p < 0.01, ^***^p < 0.001).

As shown in Figure 3, we found that treatment with liposome-encapsulated PSCT produced a significant inhibition of SARS-CoV-2 replication in the PSCT treatment group as compared to mock treatment during the first 72 h p.i. (*p* < 0.0001, by LME modeling for all time points). More specifically, at 24 h p.i., the treatment of infected Vero E6 cultures with liposome-encapsulated PSCT significantly inhibited intracellular viral replication compared to mock treatment (8.9E + 06 ± 1.3E + 06 Vs. 7.1E + 07 ± 1.3E + 07, respectively, 87.4% inhibition, *p* < 0.001 by the LME mode). This treatment also significant inhibited viral release from cells into the extracellular milieu at this time point (2.6E + 04 ± 1.5E + 04 vs. 2.1E + 05 ± 5.5E + 04, respectively, 87.8% inhibition, *p* < 0.01). The inhibition of intracellular viral replication by treatment with liposome-encapsulated PSCT was also significant at 48 h p.i. compared to mock treatment (8.6E + 08 ± 3.2E + 08 Vs. 2.5E + 09 ± 4.1E + 08, respectively, 65.2% inhibition, *p* < 0.05). Viral release from cells was also inhibited at this time point (5.3E + 06 ± 2.3E + 05 vs. 1.2E + 07 ± 1.7E + 06, respectively, 54.4% inhibition, *p* < 0.01). However, inhibition of intracellular viral replication at 72 h p.i. by liposome-encapsulated PSCT treatment was non-significant (1.7E + 09 ± 2.1E + 08 vs. 1.9E + 09 ± 5.1E + 08, respectively, *p* = N.S). The inhibition of viral release from cells by PSCT treatment at this time point was moderate yet statistically significant (3.05E + 07 ± 1.7E + 06 vs. 4.7E + 07 ± 2.2E + 06, respectively, 35.4% inhibition, p < 0.01).

### PSCT intracellular replication and release kinetics are increases in virus infected compared to uninfected cell cultures

**FIGURE 4:**
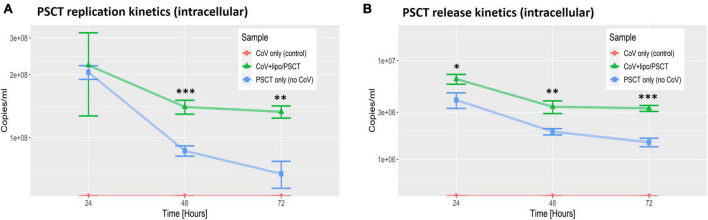
PSCT mRNA replication and release levels are dependent on co-infection with SARS-CoV-2. Vero E6 cells were either uninfected (blue line) or infected (green line) with SARS-CoV-2 viral stock at low MOI, and subsequently transfected with liposome-encapsulated PSCT (250 ng/well). Additional control cell cultures were infected but left untreated (as specified in the label). The cell lysates **(A)** and supernatants **(B)** were collected at 24, 48, and 72 h p.i., and used to determine PSCT-mRNA levels (copies/ml) by multiplex qPCR, as detailed in the methods section. The graphs demonstrate that SARS-CoV-2-infected cells treated with liposome-encapsulated PSCT displayed significantly higher intracellular replication **(A)** and extracellular release **(B)** of PSCT mRNA, as compared to the uninfected cultures at all time points (*p* < 0.01, for both analyses, by LME model). Data show the mean ± SEM of one representative experiment out of three performed in triplicates. Statistical analysis was performed by the LME model (*p < 0.05, ^**^p < 0.01, ^***^p < 0.001).

Next, we performed reciprocal analysis of PSCT-mRNA levels within cells and in the supernatants collected at 24, 48, and 72 h p.i. (Figure 4). The data demonstrated that the liposome-encapsulated PSCT entered the cells with high efficiency (> 2E + 08 copies/ml at 24 h). More importantly we found that PSCT intracellular levels were significantly higher in SARS-CoV-2 infected Vero E6 cultures as compared to counterpart PSCT-treated uninfected cultures (Figure 4), especially at 48 and 72 h p.i. (p < 0.05, by the LME model). For example, the intracellular quantity of PSCT at 48 h p.i. was significantly higher (∼1.6 fold) in infected cultures compared to uninfected cultures (1.4E + 08 ± 1.1E + 07 vs. 8.6E + 07 ± 4.9E + 06, respectively, *p* < 0.001). Furthermore, PSCT intracellular levels remained significantly higher (∼1.9 fold) in the infected cultures even at 72 h p.i. (1.3E + 08 ± 8.9E + 06 vs. 6.7E + 07 ± 9.9E + 06, respectively, *p* < 0.01).

Likewise, infected Vero E6 cells treated with liposome-encapsulated PSCT released higher numbers of PSCT particles into the extracellular milieu compared to the uninfected counterpart cultures at all time points (*p* < 0.01, by LME modeling for all time points). In detail, At 24 h p.i. PSCT release from infected cells into the medium was significantly higher as compared to control uninfected cultures (6.5E + 06 ± 7.5E + 05 vs. 4.05E + 06 ± 7.2E + 05, respectively, *p* < 0.05). Higher levels of PSCT release from the infected and treated cultures compared to the uninfected treated control cultures was even more evident at the 48 h (3.4E + 06 ± 5.01E + 05 vs. 1.9E + 06 ± 1.4E + 05, respectively, *p* < 0.01), and at 72 h time points (3.3E + 06 ± 2.3E + 05 vs. 1.5E + 06 ± 1.47E + 05, respectively, *p* < 0.001). Taken together these finding indicated that the PSCT-mRNA competes with standard SARS-CoV-2 genome for replication as well as packaging within virus infected cells.

### PSCT treatment is not associated with cellular toxicity

**FIGURE 5:**
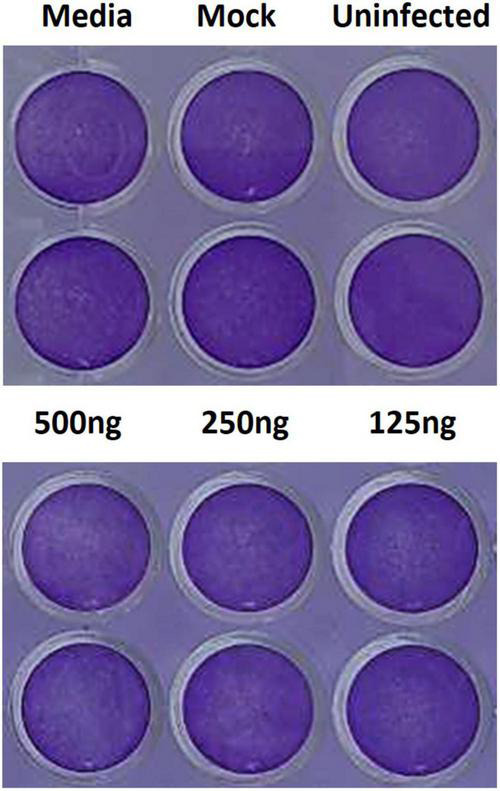
Treatment with liposome-encapsulated PSCT is not associated with evident cytotoxicity. Cell cultures were treated with liposome-encapsulated PSCT at applicable doses (125, 250, and 500 ng per well), mock-treated or untreated as indicated in the label. Then, 24 h p.i. the supernatants were removed and cells were fixed with 4% formaldehyde and stained with 0.1% crystal violet dye solution. As shown in the recorded images no substantial PSCT treatment associated cytotoxicity was observed.

To assess the cytotoxicity of the liposome-encapsulated PSCT, at 24 h p.i. the supernatants were removed and cells were fixed with 4% formaldehyde and immediately stained with 0.1% crystal violet dye solution. Cell cultures were first evaluated by optic microscopy and then recorded by digital imaging. As revealed by the recorded images, no substantial PSCT associated cytotoxicity was detected in cell cultures treated with liposome-encapsulated PSCT at relevant doses (125, 250, and 500 ng per well), as compared to mock-treated or untreated cell cultures (Figure 5). Additionally, in comparable experiments conducted for 48 h p.i. no significant apparent association between PSCT treatment and cellular toxicity was detected (data not shown).

## Discussion

**FIGURE 6:**
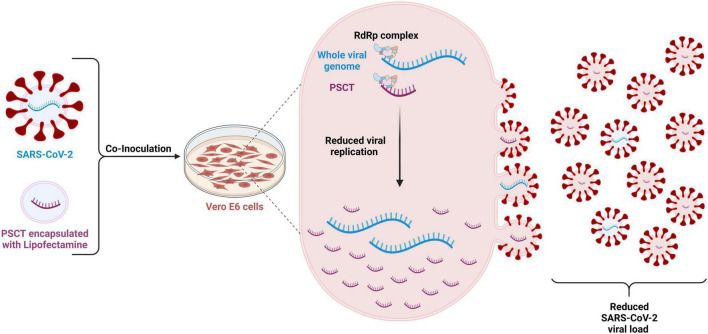
Schematic illustration of the methodology and proposed mechanism of action of the PSCT treatment. The mRNA-PSCT/liposome complexes were prepared in advance using lipofectamine-MessengerMAX™ transfection reagent as recommended by the manufacturer. Vero E6 cells were infected with SARS-CoV-2 viral stock at low MOI (0.01) for 1 h, and then the cultures were then treated once with liposome-encapsulated PSCT or with mock transfection. At 4 h post transfection, we aspirated the supernatant and replaced it with standard culture media, as detailed in methods. The supernatants and cell lysates were collected at 24, 48, and 72 h p.i., and analyzed for SARS-CoV-2 genome load and PSCT levels by multiplex qPCR. Our results imply that the liposome-encapsulated PSCT molecules were effectively delivered intracellularly and significantly inhibited the replication of SARS-CoV-2 genome by the viral replicase-transcriptase complex. Furthermore, our data regarding the release of PSCT and standard SARS-CoV-2 genome from infected cells into the extracellular milieu suggest that PSCT competed with the viral genome for packaging into viral particles. Created with BioRender.com (agreement number: MT2525A4E2).

In this study we demonstrate using the Vero E6 cell culture model of SARS-CoV-2 infection that intracellular delivery of *in silico* designed synthetic mRNA, comprised of merged key CREs positioned in a correct order, can inhibit the replication and extracellular release of the standard virus (Figure 6).

More specifically, we show that a single treatment with liposome-encapsulated PSCT of infected Vero E6 cell cultures significantly inhibited SARS-CoV-2 genome replication for up to 48 h, with peak inhibition (∼90%) at 24 h p.i. (Figure 3). Similarly, the release of virions into the extracellular milieu was significantly reduced by this treatment for up to 72 h with the maximal reduction observed at 24 h p.i. (∼87% inhibition).

In addition, we observed that liposome-encapsulated synthetic mRNA was delivered intracellularly with high efficiency (Figure 4). Importantly, we also show that intracellular levels of PSCT were maintained at higher levels in SARS-CoV-2 infected Vero E6 cultures, as compared to counterpart uninfected cultures for up to 72 h after a single treatment. The release of PSCT into the extracellular milieu was also more prominent and stable in infected cells. These observation indicates that the synthetic mRNA can compete with the standard SARS-CoV-2 for replication by the coronavirus replicase-transcriptase complex as well as for packaging into assembling nascent viral particles.

Importantly, DVGs are typically produced during the replication of RNA viruses by means of two major mechanisms, deletion and snapback/copy-back. All DVGs lack genomic regions that encode for vital viral proteins making them replication-incompetent (Pathak and Nagy, 2009; Genoyer and López, 2019). Deletion DVGs are more common in positive single-stranded RNA viruses (e.g., coronaviruses) and most likely form when the error-prone viral replicase complex drops from the RNA genome template at a “random” break point and continues replication at a distal point on another template. These deletions may be small or large but maintain genome sequences required for replication by the viral RNA polymerase (Genoyer and López, 2019). At present, it is unknown how “naturally occurring” DVGs interfere with the replication and encapsidation of the standard SARS-CoV-2 genome, and moreover their role in determining disease outcome remains uncertain. Previous studies in negative RNA viruses, particularly the influenza virus, suggest that the sequence length, intracellular production level, and the intracellular ratio of DVGs to standard viral genome load regulate interference with viral replication (Wasik et al., 2018). Moreover, there are multiple studies showing that DVGs produced during the replication of negative-sense RNA viruses have a meaningful contribution to the initiation of a robust innate immune response to the viral infection, which is uncoupled from their interference function (Yount et al., 2006; Tapia et al., 2013; López, 2014; Mura et al., 2017).

As detailed in the introduction, during the first months of the pandemic we initiated the *in silico* design of a PSCT sequence specifically engineered to include only the minimum length of key CREs required for efficient replication and packaging within cells already infected with the “helper” standard SARS-CoV-2. Our provisional patent application was submitted on April 12, 2020 (US 63/008,756). Of note, during the period of manufacturing our unique decoy synthetic mRNA and experimentally testing our hypothesis that it can interfere with SARS-CoV-2 infection in Vero E6 cells, two research reports that lend support to our findings were published (Yao et al., 2021; Chaturvedi et al., 2022). Yao et al. produced a 2882 nucleotide-long DVG of SARS-CoV-2, combining regions of the viral genome that do not encode for functional proteins yet may replicate and undergo packaging (Yao et al., 2021). Next, the researchers transfected Vero-E6 cells, by electroporation with their DVG produced by IVT, at 1 hour prior to infecting the cells with the WT SARS-CoV-2. They found that this pre-treatment reduced the viral load in infected cells by half during the first 24 hours of infection. Moreover, their DVG replicated and was transmitted as efficiently as the full-length genome. A possible explanation for the more potent inhibitory effect of the PSCT treatment observed in our *in vitro* experiments is that the sequence length of their DVG was approximately two-fold longer compared to the PSCT sequence length.

More recently, Weinberger and colleagues (Chaturvedi et al., 2021) published their studies showing a proof of concept for the therapeutic potential of interfering DVGs, which they termed therapeutic interfering particles (TIPs). They first showed that transfection with TIP1 and TIP2 of infected Vero E6 cells produced significant inhibition of SARS-CoV-2 replication at 24, 48, and 72 h p.i. Moreover, by using a VLP system and subsequent imaging by transmission electron microscopy, they provide data suggesting that TIP1 and TIP2 can induce the release of VLPs from the cells. Next, using a hamster model of SARS-CoV-2 infection, they provide evidence that single intranasal administration of TIP1 encapsulated in lipid nanoparticles significantly reduces viral load in the lungs and certain disease outcomes (e.g., lung inflammation/infiltration and edema). Of note, the TIP1 (∼2.1 kb length) encoded only two combined sections from SARS-CoV-2 genome, the first 450nt of the 5′ UTR plus a small part of ORF1ab and the last 328nt of the 3′-end. TIP2 (∼3.5 kb length) encoded 1,540nt encompassing the 5′UTR and part of ORF1ab joined to the last 713nt of the 3′-end of the viral genome. Accordingly, it seems that the sequence of TIP1/2 did not include a *’bona fide’* PS, previously postulated to be positioned at the 3-end of ORF1ab and to facilitate effective genome packaging (Hsieh et al., 2005; Masters, 2019).

In contrast, our PSCT sequence contained three joined regions of the viral standard genome as follows: the extended 5′UTR (positions 1–475), followed by our *in silico* predicted PS from the 3-end of ORF1ab (positions 19,657-20,384), and a highly conserved 3′-end sequence (positions 29,603-29,870). In this regard, in a recent *Science* paper, the researchers determined the optimal SARS-CoV-2 cis-acting RNA sequence important for efficient genome packaging (Syed et al., 2021). They generated a VLP-based experimental system and sequentially screened 28 overlapping tiled segments (T1 to T28) from the SARS-CoV-2 genome for their capacity to act as an efficient PS. Importantly, they determined that the most efficient PS was located within the T20 segment (positions 20,080–22,222) located near the 3′ end of ORF1ab, which partially but not fully overlapped with our *in silico* predicted PS580-corresponding sequence (nucleotides 19,675–20,348). The genomic sections of SARS-CoV-2 combined in TIP1/2 have no overlap with the PS-containing T20 section.

## Conclusion and future directions

In summary, our findings show that an *in silico* designed synthetic mRNAs, pre-engineered to include only “optimal minimum length” key CREs from SARS-CoV-2, can interfere with the replication and extracellular release of WT SARS-CoV-2 in the classical Vero E6 cell-based infection model. These results also imply a translational potential for similar *in silico* designed mRNA compounds that could interfere with the replication and packaging of other ssRNA viruses that share genome organization with the *Coronaviridae*. This concept should be further tested in various prophylactic and therapeutic preclinical animal models of SARS-CoV-2 and other pathogenic zoonotic ssRNA virus infections. Of significant relevance to the translational horizon of this novel technology are the ongoing global scientific efforts to develop lipid nanoparticles (LNPs) with complex structures, aimed to overcome relevant biological barriers and deliver mRNA-based therapeutics into the precise target tissue (Tenchov et al., 2021). We predict that the recent successful development of LNPs as the delivery vehicle for the mRNA-based vaccines against SARS-CoV-2 together with improved LNP formulations that facilitate tissue-specific delivery of “interfering” mRNAs (intranasal, intra-bronchial, transdermal, oral/sublingual or parenteral) should accelerate the adoption of this novel anti-viral therapy.

## Patent disclosures

The Sheba Medical Center submitted a patent application that describes the PSCT composite and the methodology. The international patent publication number is WO2021/209984A1 (Priority data: US63/008,756 submitted 12.4.2020).

## Data availability statement

The original contributions presented in this study are included in the article/Supplementary material, further inquiries can be directed to the corresponding author. The PSCT sequence is available at GenBank accession number: OQ548088.

## Author contributions

EG, MM, and IG: study conception and design. NA, HA, and OE: acquisition of data. NA, OE, YS, MM, and IG: analysis and interpretation of data. NA, EG, MM, and IG: writing. MM and IG: supervision. All authors were involved in drafting the article or revising it critically for important intellectual content, and approved the final version to be published.

## Footnotes

1
https://covid19.who.int/data

2
https://www.bioline.com/sensifast-probe-no-rox-one-step-kit.html

3
https://genome.ucsc.edu/cgi-bin/hgBlat?command=start
