# Supplementary material for: Proof-of-concept for effective antiviral activity of an in silico designed decoy synthetic mRNA against SARS-CoV-2 in the Vero E6 cell-based infection model
Source: Front Microbiol. 2023 Apr 20;14:1113697. doi: 10.3389/fmicb.2023.1113697 (PMC10157240; doi:10.3389/fmicb.2023.1113697)
Supplement: Supplementary file 2 [file Table_1.DOCX]

**Supplemental Table 1.** List of the 10 different SARS-like coronavirus strains used in the multiple alignment analysis as depicted in Supplemental Fig. 1.

| No. | GenBank Accession | Descriptive name |
| --- | --- | --- |
| **1** | **NC045512** | SARS-CoV-2/Wuhan-Hu-1 |
| **2** | **AY291451** | SARS coronavirus TW1 |
| **3** | **DQ022305** | Bat SARS CoV HKU3-1 |
| **2** | **DQ412043** | Bat SARS coronavirus Rm1 |
| **4** | **GQ153542** | Bat SARS CoV HKU3-7 |
| **5** | **KY417147** | Bat SARS-like CoV Rs4237 |
| **6** | **JX993987** | Bat CoV Rp/Shaanxi2011 |
| **7** | **JX993988** | Bat CoV Cp/Yunnan2011 |
| **8** | **KJ473814** | BtRs-BetaCoV/HuB2013 |
| **9** | **GQ153547** | Bat SARS CoV HKU3-12 |
| **10** | **MN996532** | Bat CoV RaTG13 |
