# Supplementary figures and images for: Proof-of-concept for effective antiviral activity of an in silico designed decoy synthetic mRNA against SARS-CoV-2 in the Vero E6 cell-based infection model
Source: Front Microbiol. 2023 Apr 20;14:1113697. doi: 10.3389/fmicb.2023.1113697 (PMC10157240; doi:10.3389/fmicb.2023.1113697)

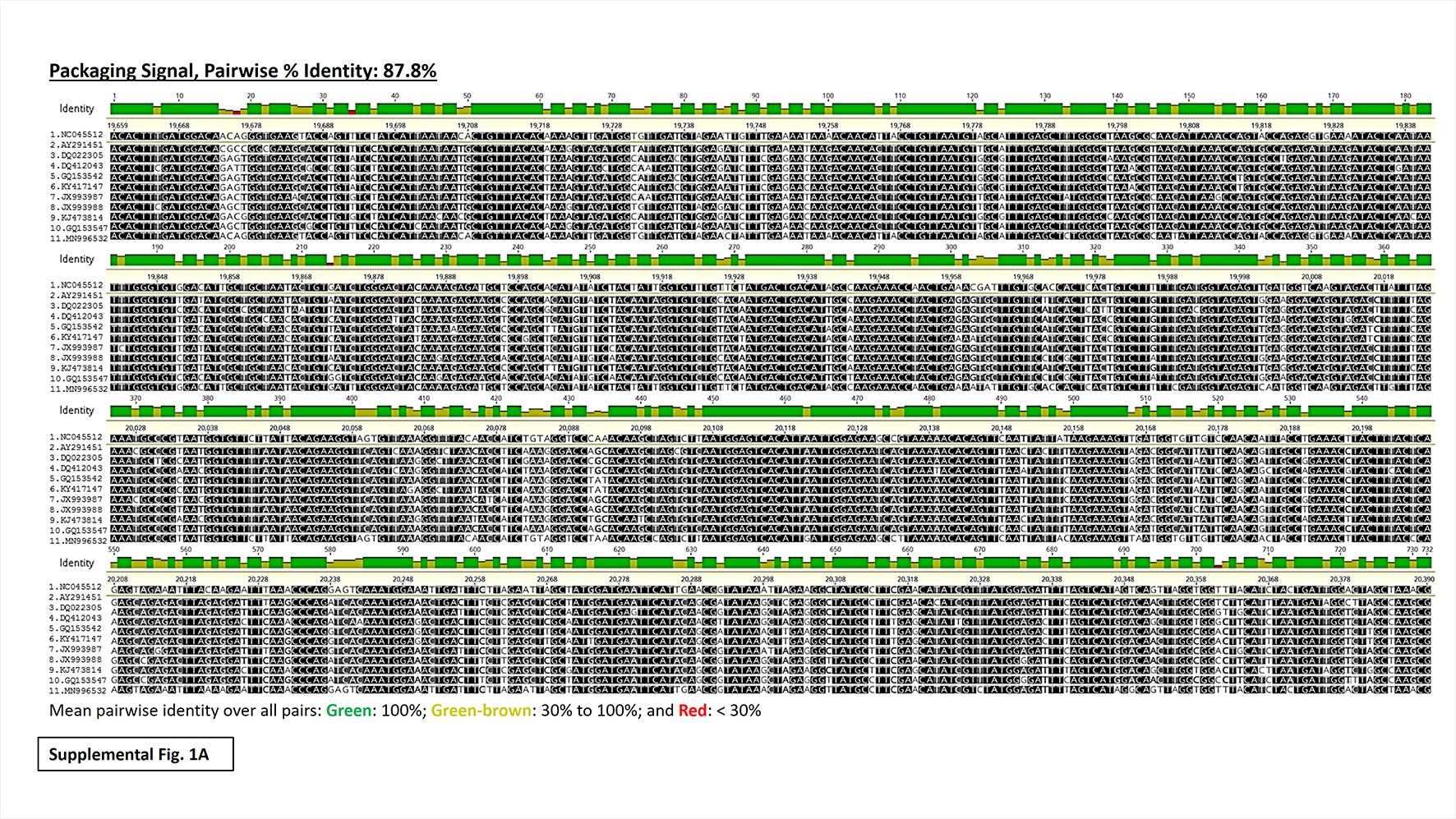

Supplement: Supplementary file 3 [file Image_1.TIF]

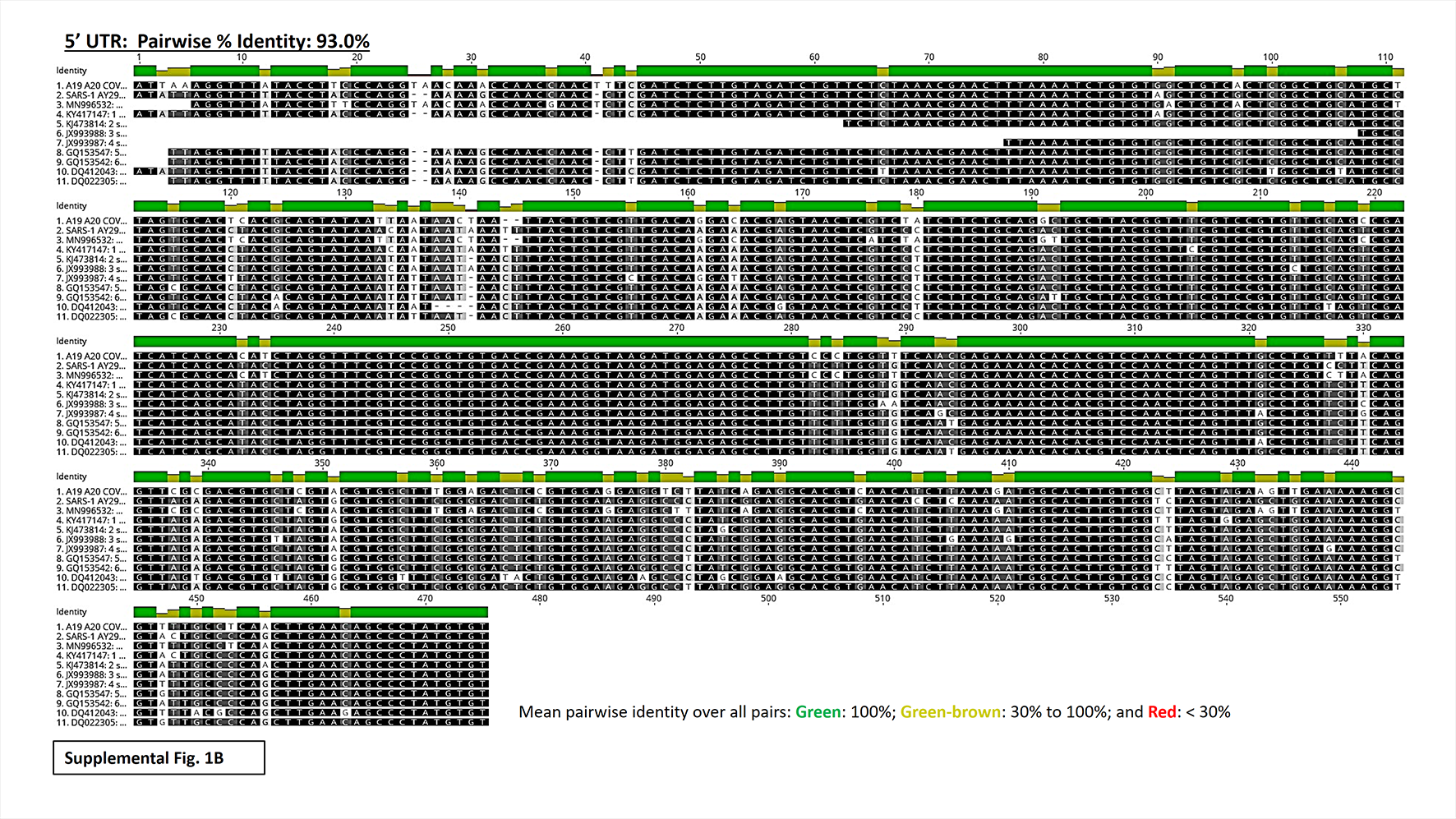

Supplement: Supplementary file 4 [file Image_2.TIF]

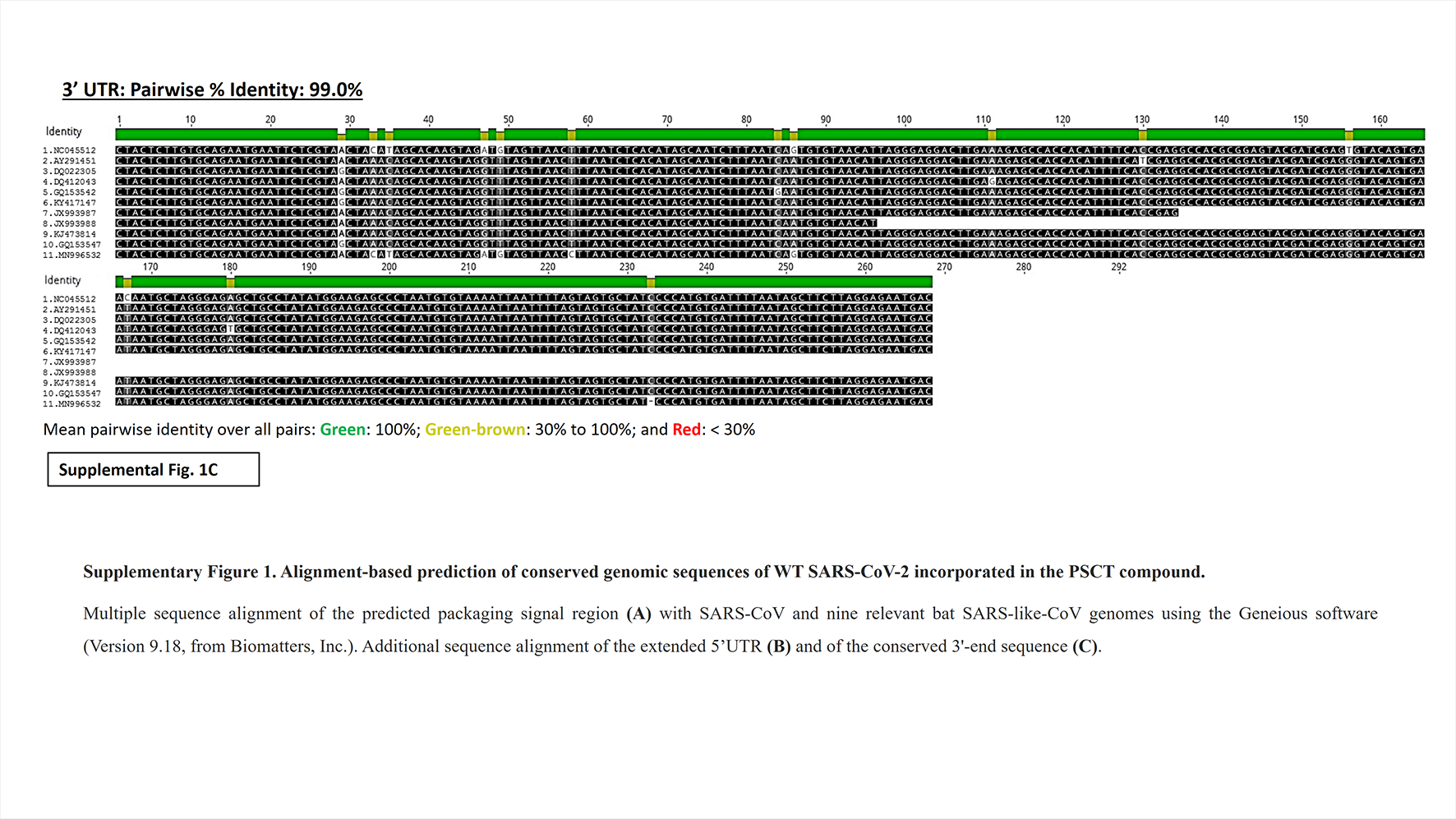

Supplement: Supplementary file 5 [file Image_3.TIF]
